# Transcriptional changes during hepatic ischemia-reperfusion in the rat

**DOI:** 10.1371/journal.pone.0227038

**Published:** 2019-12-31

**Authors:** Valerie Zabala, Joan M. Boylan, Paul Thevenot, Anderson Frank, Dewahar Senthoor, Varun Iyengar, Hannah Kim, Ari Cohen, Philip A. Gruppuso, Jennifer A. Sanders

**Affiliations:** 1 Department of Pediatrics, Rhode Island Hospital and Brown University, Providence, RI, United States of America; 2 Division of Biology and Medicine, Brown University, Providence, RI, United States of America; 3 Institute of Translational Research, Ochsner Health Systems, New Orleans LA, United States of America; 4 Warren Alpert Medical School, Providence, RI, United States of America; 5 Department of Molecular Biology, Cell Biology and Biochemistry, Brown University, Providence, RI, United States of America; 6 Department of Pathology and Laboratory Medicine, Brown University, Providence, RI, United States of America; National Institutes of Health, UNITED STATES

## Abstract

There are few effective targeted strategies to reduce hepatic ischemia-reperfusion (IR) injury, a contributor to poor outcomes in liver transplantation recipients. It has been proposed that IR injury is driven by the generation of reactive oxygen species (ROS). However, recent studies implicate other mediators of the injury response, including mitochondrial metabolic dysfunction. We examined changes in global gene expression after transient hepatic ischemia and at several early reperfusion times to identify potential targets that could be used to protect against IR injury. Male Wistar rats were subjected to 30 minutes of 70% partial warm ischemia followed by 0, 0.5, 2, or 6 hours of reperfusion. RNA was extracted from the reperfused and non-ischemic lobes at each time point for microarray analysis. Identification of differentially expressed genes and pathway analysis were used to characterize IR-induced changes in the hepatic transcriptome. Changes in the reperfused lobes were specific to the various reperfusion times. We made the unexpected observation that many of these changes were also present in tissue from the paired non-ischemic lobes. However, the earliest reperfusion time, 30 minutes, showed a marked increase in the expression of a set of immediate-early genes (c-Fos, c-Jun, Atf3, Egr1) that was exclusive to the reperfused lobe. We interpreted these results as indicating that this early response represented a tissue autonomous response to reperfusion. In contrast, the changes that occurred in both the reperfused and non-ischemic lobes were interpreted as indicating a non-autonomous response resulting from hemodynamic changes and/or circulating factors. These tissue autonomous and non-autonomous responses may serve as targets to ameliorate IR injury.

## Introduction

Ischemia-reperfusion (IR) injury is an unavoidable surgical complication that can lead to early graft dysfunction following liver transplantation. In the United States, nearly 40% of patients that entered the liver donor waiting list in 2013 still had not received a transplant within a year, and almost 20% remained on the list in 2017 [1]. This is due, in part, to the shortage of viable livers that can withstand IR injury and be successfully transplanted. Donor livers that do not meet the criteria for transplantation, often due to age or steatosis, are discarded [2, 3]. These circumstances indicate a need for pharmacological therapies that could promote graft survival of marginal livers, thereby increasing the donor pool and reducing patient morbidity.

The IR injury cascade is divided into three stages: ischemia, early reperfusion, and late reperfusion. The duration of ischemia impacts the severity of injury, which begins immediately upon reintroduction of blood and the resulting generation of excess reactive oxygen species (ROS) [4]. The ROS generated at the onset of reperfusion result in both direct cellular damage (necrosis, membrane disruptions) and indirect damage through cellular signaling that induces an inflammatory response [5, 6]. This leads to a biphasic progression of injury. The early phase is characterized by ROS production, parenchyma dysfunction and secretion of pro-inflammatory cytokines, including TNFα and IL-6 [7]. These events lead to the late reperfusion phase, characterized by neutrophil infiltration, which results in further ROS generation, thus compounding hepatic damage [8].

Most therapeutic strategies currently under investigation to attenuate IR injury employ pharmacological agents to disrupt the reperfusion injury cascade [9]. These include rescuing cells from ROS-induced damage by administration of antioxidants, enhancing the clearance of ROS-induced cellular damage through promotion of autophagy, or dampening of the immune response [10–12]. Recent advances in our understanding of hepatic IR injury have revealed mechanisms outside of the well-accepted models of hypoxia and inflammation that contribute to injury progression [13, 14].

Ischemic preconditioning (IPC), a nonpharmacological intervention, has shown promise in achieving a reduction of hepatic IR injury [15]. During IPC, the liver is subjected to short bursts of ischemia before prolonged ischemia. While IPC has been shown to ameliorate liver injury in small animal models, translation to clinical practice has produced mixed results [16–19]. These inconsistent results may be due to variation in IPC protocols. Furthermore, the design of IPC protocols suffers from a fundamental lack of understanding of the mechanisms by which IPC exerts its protective effects. The hormetic effect of ischemia-reperfusion likely involves multiple upstream effectors and responses. A greater understanding of the cellular response to IPC could lead to optimized protocols and targeted approaches to promote hepatocyte survival and preserve liver function during resection and transplantation.

In the present study, we used a rat model of thirty minutes of 70% partial, warm ischemia to investigate changes in global gene expression at multiple time points during early reperfusion [20]. This brief period of ischemia was selected to study changes in the absence of overt hepatic damage. We profiled changes in gene expression and performed pathway analyses to gain insight into molecular mechanisms involved in this sub-injury response. To discern changes in the transcriptome exclusive to IR, we compared changes in gene expression of the reperfused lobes to those lobes that did not experience blood interruption (heretofore referred to as the non-ischemic lobes). We did so to use the non-ischemic tissue as an additional control condition. However, we found that many of the changes that occurred in the reperfused lobe were mirrored in the non-ischemic lobe, indicating the contribution of hemodynamic and/or circulating factors to the IR response in this well-defined and widely used rodent IR model.

## Materials and methods

### Animals

Male Wistar rats (5–6 weeks) were purchased from Charles River Laboratories (Wilmington, MA) and group housed in pairs in a conventional room under standard conditions (mixed paper and cellulose bedding, Nylan bone enrichment, 12 hr light/dark cycle, temperature maintained at 70°F). Rats had free access to food (Teklad global 18% protein diet) and autoclaved water.

**Fig 1:**
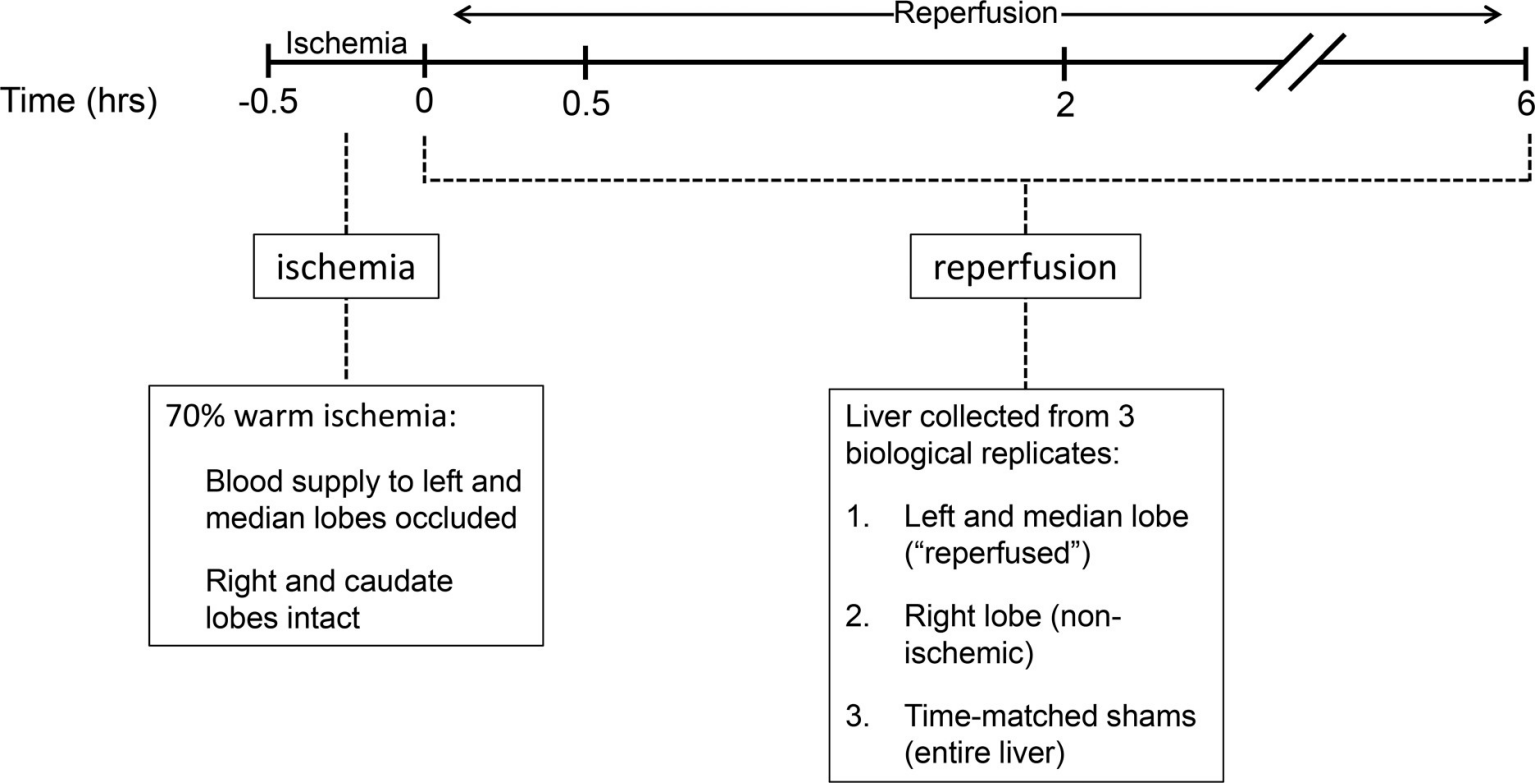
Rat model of 30 minutes of warm 70% ischemia with various reperfusion times. Male Wistar rats (n≥4) were separated into 4 groups based on reperfusion time: 0 hr (no reperfusion), 0.5 hour, 2 hours, and 6 hours. All groups received 30 minutes of warm ischemia by occlusion of blood flow to the left and median lobes (70% of liver). Blood flow to the right and caudate lobes (30% of liver) was not interrupted. Triplicate sham-operated animals were matched at each time point. Left and median lobes are labeled as “reperfused” and right lobes as “non-ischemic.” Randomly chosen triplicate biological replicates from each experimental group and reperfusion time were used for microarray analyses.

A model of 70% warm ischemia followed by reperfusion (Fig 1) was used to generate the following four groups (n≥4): 0 hr (no reperfusion); 0.5 hr reperfusion; 2 hr reperfusion; 6 hr reperfusion; 3 sham-operated animals per corresponding time point. All surgeries were performed mid-morning under isoflurane anesthesia as previously described [21]. Briefly, the abdominal cavity was exposed by a midline incision and the hepatoduodenal ligament (hepatic artery, portal vein, and bile duct) to the left and median lobes was occluded for 30 min using a reversible clamp. This model of warm 70% ischemia and reperfusion was visually confirmed by the blanching and return of the color to the left and median lobes and continuous perfusion of the right and caudate lobes. Sham animals at each time point underwent liver manipulation to mimic the application and removal of an ischemic clamp without blood interruption. Lidocaine was applied to the skin incision. All animals were euthanized by exsanguination under isoflurane anesthesia. All animal studies were performed in accordance with the guidelines of the National Institutes of Health and approved by the Oschner Animal Care and Use Committee.

### AST/ALT measurements and histological analysis

At each reperfusion time, blood was collected by cardiac puncture and liver was harvested. Sections of each liver lobe (left, medial,right) were either fixed in 10% neutral buffered formalin for subsequent paraffin embedding, or flash frozen in liquid nitrogen and subsequently stored at -80°C. Formalin fixed, paraffin embedded tissues were sectioned (7 μm), stained with hematoxylin and eosin, and scored blindly by Midwest Veterinary Pathology (Lafayette, IN) using a modified Suzuki score as described previously [22]. Serum collected by centrifugation from blood at the time of exsanguination was snap-frozen and stored at -80°C. Serum levels of aspartate aminotransferase (AST) and alanine aminotransferase (ALT) were measured by the Lifespan Pathology Core Facility (Providence, RI) using standard methods on a Beckman Coulter AU 5800 analyzer. AST/ALT measurements at each reperfusion time were compared to all sham animals using a Mann-Whitney test followed by Bonferroni correction. These statistical analyses were performed in GraphPad Prism (San Diego, CA) [23].

### Microarray analysis

RNA was isolated from the right (non-ischemic) and combined left and medial lobes (reperfused) from randomly chosen triplicate animals at each reperfusion time using the Qiagen RNeasy Mini Kit (QIAGEN, Inc., Germantown, MD) as per the manufacturer's instructions.

Integrity of the purified RNA was assessed using the Agilent 2100 Bioanalyzer before analysis using Affymetrix Rat Transcriptome 1.0 arrays (Affymetrix, Santa Clara, CA). Raw expression data were normalized by Signal Space Transformation in conjunction with Robust Multivariate Analysis (SST-RMA) using the Affymetrix Expression Console. Quality control analysis was performed before the data were exported with annotated probe sets. Annotated, normalized probe sets corresponding to coding genes were used for all gene expression analyses. All microarray data were deposited into NCBI’s Gene Expression Omnibus (GEO, http://www.ncbi.nlm.nih.gov/geo) and are available using GEO Series accession number 117915.

### Gene expression and pathway analyses

Principal component analysis (PCA) was performed in R using the *prcomp* function. Results were displayed as a three-dimensional plot [24]. To perform ANOVA, probe sets were first filtered using the median interquartile range (IQR) of all values as a threshold before using the *limma* Bioconductor package [25]. Pair-wise comparisons were made between the reperfused and non-ischemic lobes at each time point, as well as to the group of shams pooled from all 4 time points. P-values were adjusted for the False Discovery Rate (FDR), and resulting q-values less than 0.05 considered significant. To display differentially expressed genes, volcano plots were constructed with the log_2_ fold change and the -log_10_ q-value of each probe set in each comparison. Ingenuity Pathway Analysis (IPA®), used for pair-wise comparisons, was conducted using only statistically significant probe sets (QIAGEN Inc., https://www.qiagenbioinformatics.com/products/ingenuity-pathway-analysis) [26]. Results with p-values <10^−7^ were considered significant based on p-values generated from 5 similarly sized sets of randomly selected genes with a fold-change in expression ≤ 1.1 [27, 28].

### Reverse transcription and qPCR

Total RNA from frozen liver tissue of biological triplicates was extracted using the Qiagen RNeasy Mini Kit (QIAGEN, Inc., Germantown, MD). Each lobe (non-ischemic and reperfused) was processed separately. An RNA concentration of 0.5 ug was used for cDNA synthesis using the iScript Reverse Transcription Supermix (BioRad, Hercules, CA). Levels of mRNA were detected with the SsoAdvanced Universial SYBR Green Supermix (BioRad, Hercules, CA) on a BioRad c1000 Touch Thermal Cycler. Paired, validated rat primers for Fos, Fgf21, Cyr61 and 18s rRNA (PrimePCR, BioRad, Hercules, CA) were used. Data were generated using BioRad CFX Manager 3.1 (BioRad, Hercules, CA).

### Signosis transcription factor arrays

Transcription Factor (TF) activity was assessed using the TF Activation Profiling Plate Array II (Signosis Inc., Santa Clara, CA). We analyzed nuclear extracts isolated from triplicate samples of the reperfused lobe after 30 minutes of reperfusion and corresponding sham controls. Each array hybridizes samples to 96 TF-bound probe complexes (95 TF’s with 1 negative control), using luminescence for quantification of TF activity. Each plate was normalized to the control sample and relative fluorescence was recorded.

### Western immunoblotting

Protein was extracted from frozen liver tissue from the 0.5hr reperfusion time point and sham-operated animals. Each lobe (non-ischemic and reperfused) was processed separately (n = 4), except for the sham group. Protein concentrations were measured using Thermo Scientific Pierce BCA Protein Assay (Thermo Scientific Franklin, MA). The total amount of protein loaded for each set of antibodies is as follows: S6 (30 μg), AMPK (40 μg), ERK (80 μg), JNK (80 μg), and p38 (80 μg). After semi-dry transfer to PVDF membranes, blots were blocked in 5% milk/TBST for 1 hour at 37°C and incubated overnight with primary antibody. Antibodies to phospho- ribosomal protein S6 (pS6) S235/S236 (#2211S), ribosomal protein S6 (#2217), phospho- AMP*-*activated protein kinase α (pAMPKα) T172 (#2535), AMPKα (#2532), phospho- extracellular signal–regulated kinase 1/2 (pERK 1/2) T202/Y204 (#4370), phospho- c-Jun N-terminal kinase (pJNK) T183/Y185 (#4668), JNK (#9252), phospho-p38 mitogen-activated protein kinase (p-p38) T180/Y182 (#4511), p38 (#8690) and GAPDH (#5174) were from Cell Signaling Technology (Danvers, MA). ERK 1/2 antibody (#06–182) was from Millipore-Sigma (Burlington, MA). All blots were incubated with anti-rabbit secondary (GE Healthcare, Marlborough, MA). Five ml of ECL Prime (GE Healthcare, Marlborough, MA) was added to each blot for 5 minutes before multiple exposures were acquired using the ChemiDoc-It imaging system (UVP, Upland, CA). The total blot for each signaling protein was stripped and re-probed as described above using an antibody against GAPDH.

### Kinexus antibody microarray analysis

Chemically-cleaved protein homogenates from triplicate samples of reperfused and non-ischemic lobes harvested after 2 hr of reperfusion were prepared for Kinexus KAM 900P antibody microarrays per the manufacturer instructions (Vancouver, British Columbia, Canada). Microarray results for 613 phosphosite-specific and 265 pan-specific antibodies were normalized by protein content of each lysate. Results with a q-value less than 0.05 by ANOVA (*limma*, Bioconductor) were considered statistically significant.

### Additional statistical analyses

For RT-qPCR studies, relative mRNA expression was calculated for each gene of interest using the comparative CT method with *18s* as the reference. Fold change was calculated as the ratio of the reperfused to the non-ischemic lobe for each animal. For the Signosis transcription factor array results, transcription factors that changed significantly in the 0.5 hr reperfused lobes were identified by one-way ANOVA comparisons to a sham group.

Densitometric analysis of western blots was carried out using the VisionWorks LS Analysis Software (Analytik Jena US LLC, Upland, CA). Raw data was exported to GraphPad Prism (GraphPad Software, La Jolla, CA) for statistical analyses. For each antibody probe, the protein bands were normalized to control band on the blot and expressed as a ratio. For phospho-specific immunoblotting, the normalized phospho-band was divided by the normalized total protein band in order to reflect the stoichiometry of phosphorylation. These data were then used for a paired t-test (non-ischemic to reperfused lobe) in Prism.

## Results

### Thirty minutes of warm 70% ischemia followed by reperfusion is a model of transient injury without significant hepatocyte necrosis

**Fig 2:**
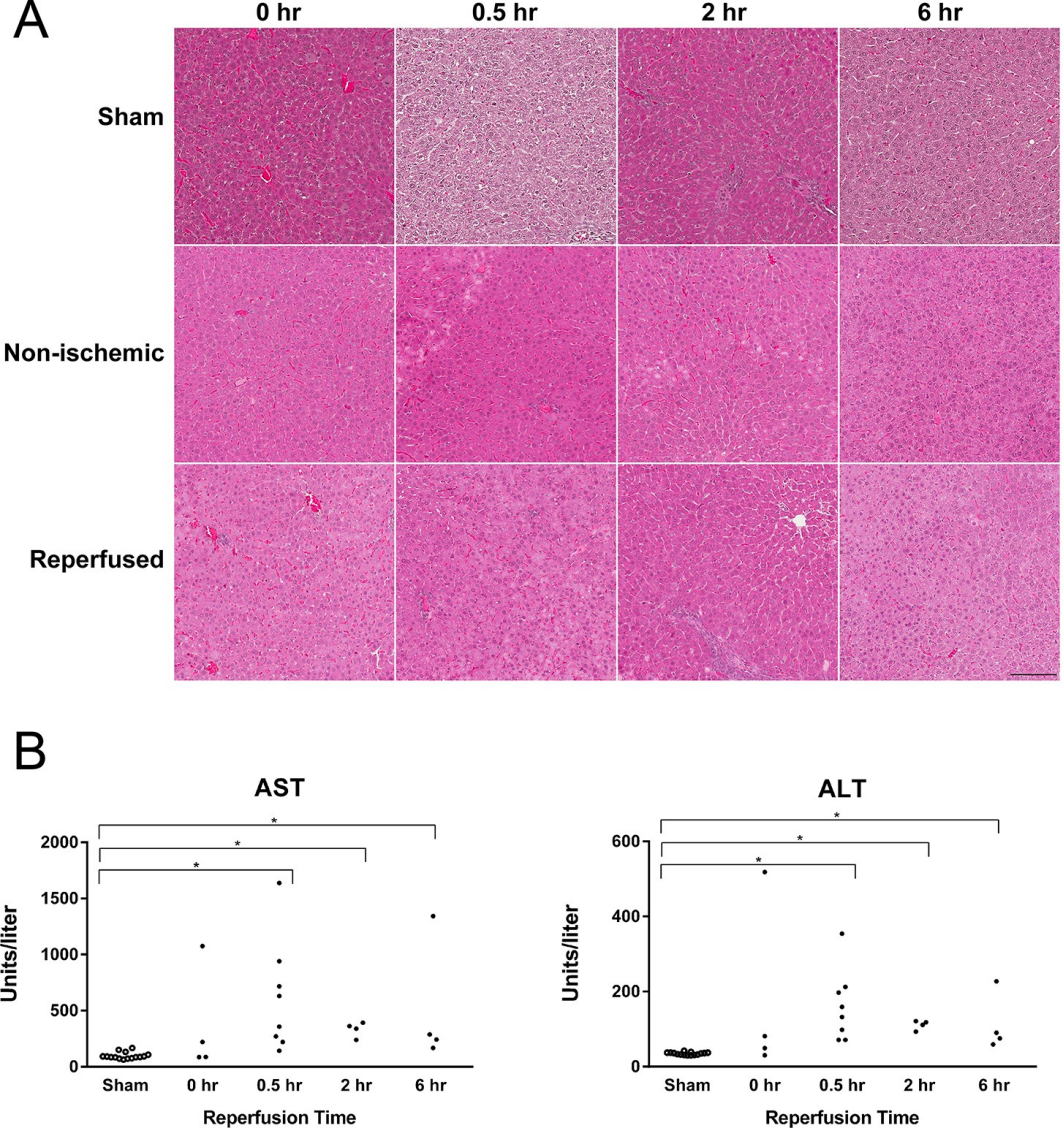
Histology and liver enzyme analysis from rats subjected to 70% warm hepatic ischemia-reperfusion. (A) Formalin-fixed and paraffin-embedded liver sections of paired non-ischemic and reperfused lobes at each reperfusion time, as well as corresponding sham-operated animals, were stained with hematoxylin and eosin (H&E). Blinded histological analysis showed no significant changes in liver architecture, sinusoidal congestion, hepatocyte vacuolization, or hepatic necrosis in the reperfused compared to non-ischemic lobes at any time point. Representative images were captured at 20x. The scale bar represents 150 μm. (B) Serum concentrations of aspartate aminotransferase (AST) and alanine aminotransferase (ALT) for individual animals in all groups are represented as individual points. Samples from sham-operated animals from each timepoint were pooled to yield a total of 12 samples (3 per experimental group). Significant changes in groups compared to shams were determined by a Mann-Whitney test followed by Bonferroni correction (*p<0.001).

We used a model of 70% warm ischemia followed by 4 reperfusion times (0, 0.5, 2, and 6 hours) to examine cellular events after brief IR (Fig 1). To assess the degree of hepatic injury in our model, we analyzed serum and processed liver samples for histology. Examination of H&E-stained sections from reperfused lobes (left and median) and non-ischemic lobes (right) at each time point showed no changes in liver architecture when compared to each other or to sections from sham-operated animals (Fig 2A). Small numbers of necrotic cells were observed in the reperfused and non-ischemic lobes compared to time-matched shams. However, no significant changes in sinusoidal congestion, hepatocyte vacuolization, or hepatic necrosis were noted in the reperfused compared to non-ischemic lobes at any of the time points examined (S1 Table). After 30 minutes of reperfusion, there was an increase in AST and ALT levels compared to that found in samples from sham animals from all four time points (10.1- and 6.7-fold, respectively; Fig 2B). Small increases were also observed in the 2 hr and 6 hr groups compared to sham. We interpreted these data as confirmation that this IR model results in minimal, transient liver dysfunction without evidence of persistent cellular or tissue damage.

### Gene expression profiles of the reperfused and non-ischemic lobes are similar at each reperfusion time

**Fig 3:**
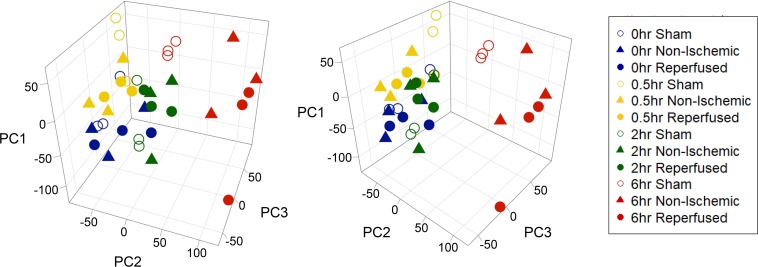
Principal component analysis (PCA) of reperfused, non-ischemic, and sham-operated groups at all reperfusion times. Two perspectives of the same 3-dimentional plot are shown. Probe sets (23,409) corresponding to coding genes were used. The first, second, and third principal components (PC1, PC2, PC3) of each sample were represented 14.7%, 11.1% and 7.9% of the variance, respectively. Visualization of the data reveals clustering of groups based on time but not condition (reperfused vs non-ischemic). The 6 hr samples (shown in red) cluster together but apart from all other times. The color of the symbols corresponds to reperfusion time (blue, 0 hr; yellow, 0.5 hr; green, 2 hr; red, 6 hr). Symbol shape indicates the experimental condition (solid circles, reperfused; solid triangles, non-ischemic; unfilled circles, shams).

There is limited literature on changes in global gene expression that occur during transient hepatic ischemia and early reperfusion times (up to 6 hr). To identify changes in the liver transcriptome associated with ischemia-reperfusion, RNA microarrays from randomly chosen biological triplicates for each group were analyzed. We first performed principal component analysis (PCA) [29] to examine the gene expression relationships between the reperfused and non-ischemic lobes at each time. PCA was performed on all probe sets that represented coding genes (23,409), and the first three components were plotted for data visualization (Fig 3). In contrast to our expected results, we found clustering of the reperfused and non-ischemic lobes at each time point. Reperfused and non-ischemic lobes from the first three time points (0, 0.5, and 2 hr) clustered together, while both conditions after 6 hr of reperfusion separated from the other groups. The sham-operated groups were clustered near the early time points except for the 6 hr shams, which were intermediate between the early and 6hr points.

**Fig 4:**
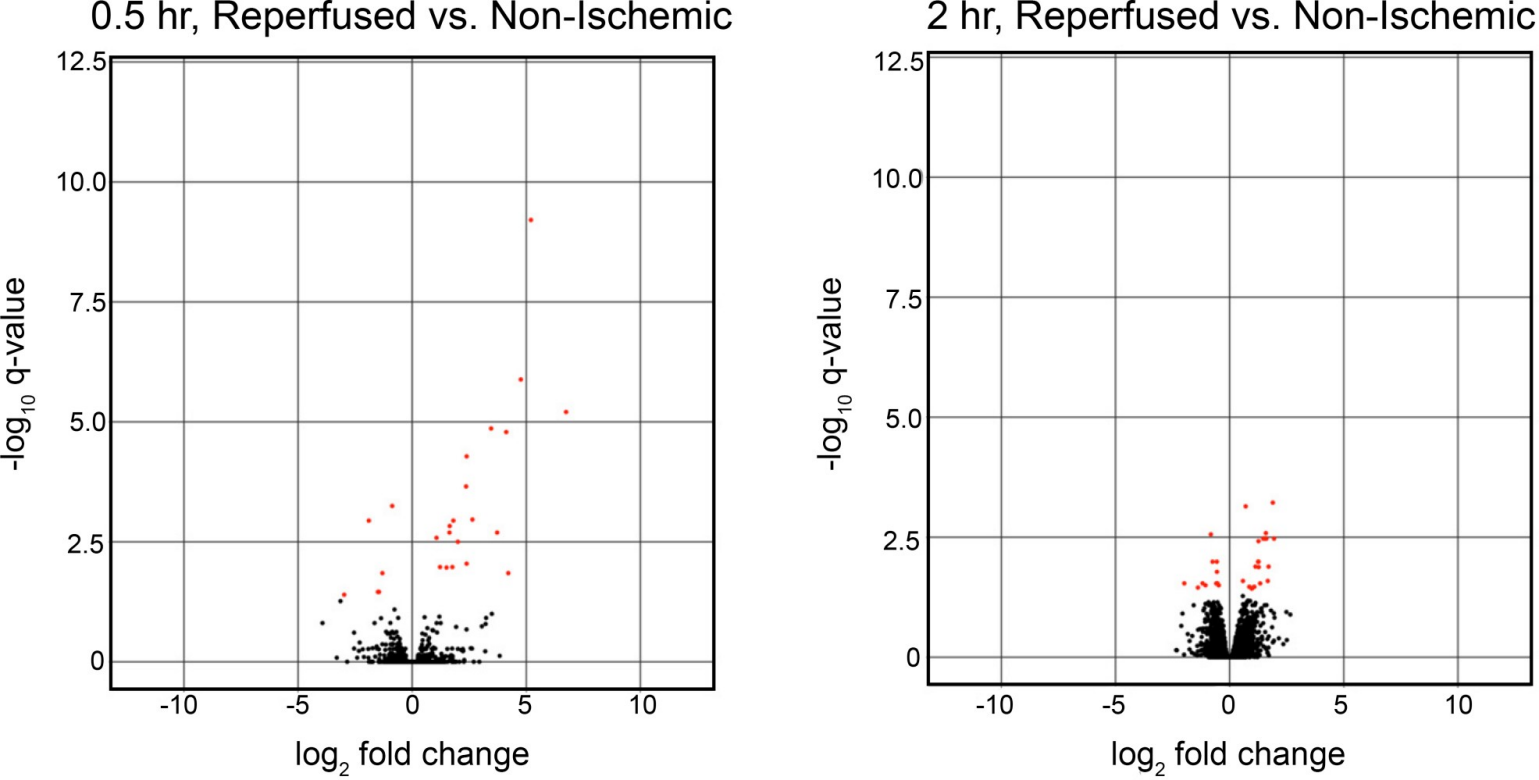
Volcano plots of pairwise comparisons of reperfused and non-ischemic lobes following 0.5 and 2 hr of reperfusion. To identify differentially expressed genes, filtered probe sets corresponding to coding genes (11,704) were subjected to one-way ANOVA. The comparisons were pairwise for the reperfused and non-ischemic lobes at each time point. No probesets were found to change significantly in the 0 hr and 6 hr comparisons. Results are graphed as volcano plots where the x-axis is the log_2_ fold change in expression and the y-axis is the -log_10_ q-value for each probe set comprison. Statistically significant probe sets (q<0.05) are highlighted in red.

To identify probe sets whose expression changed significantly, we performed pairwise ANOVA to compare the reperfused and non-ischemic lobes at each time point to each other and to pooled sham-operated animals (S2 Table). We visualized the resulting differentially expressed probe sets using volcano plots. In the comparison of the reperfused lobes to their paired non-ischemic counterpart (Fig 4), no probe sets were found to change significantly at the 0 hr or 6 hr time points (S2 Table). There were 24 and 31 probe sets that changed significantly in the 0.5 and 2 hour comparisons, respectively. The 24 probesets in the 0.5 hr comparison were mostly upregulated in the reperfused lobe and included immediate early genes (Fos, Jun, Ier2, Egr2, Atf3). The fold-change of the probe sets found to be significantly altered in the 2 hr comparison were considerably smaller than those found in the 0.5 hr comparison.

**Fig 5:**
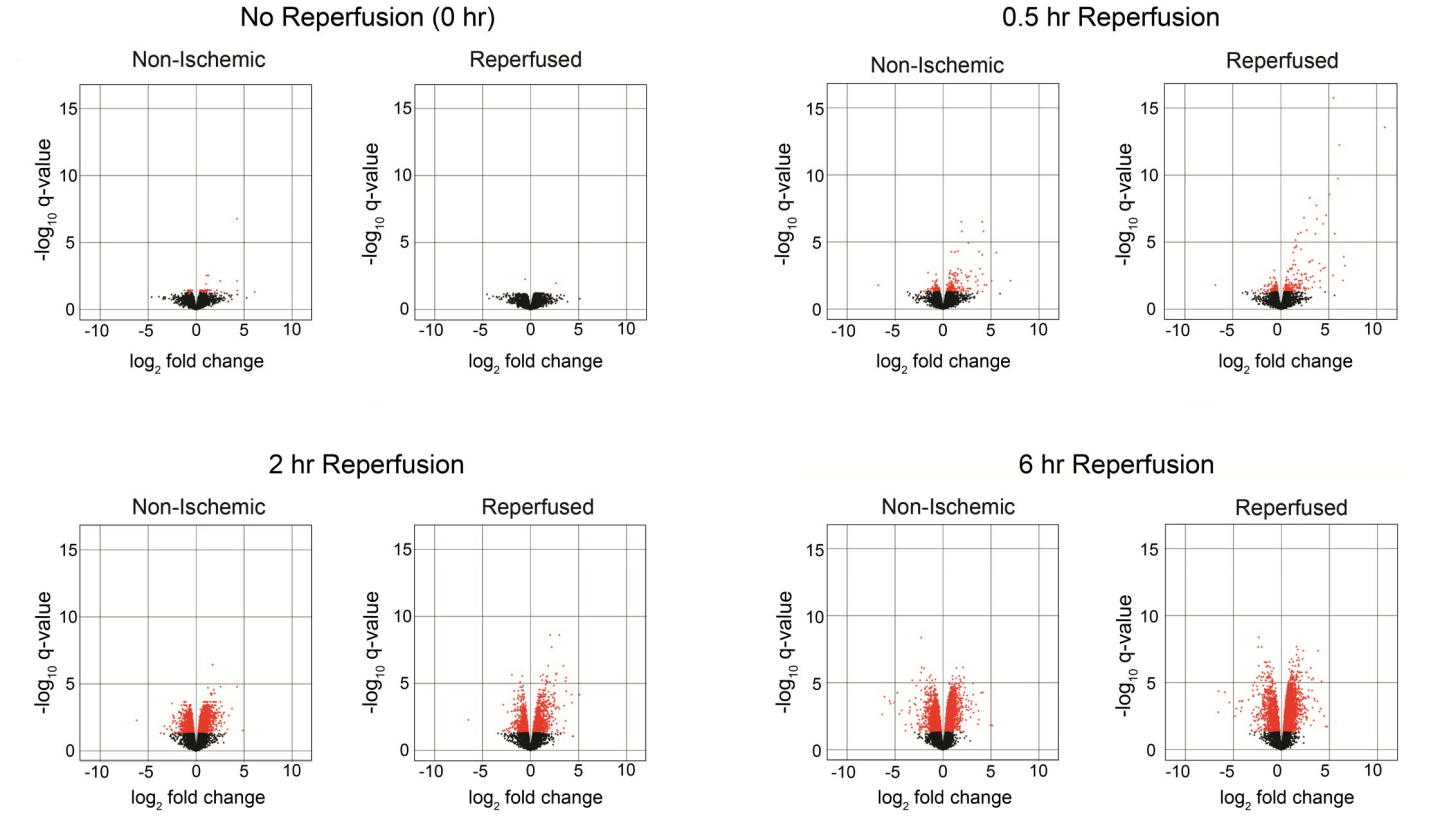
Volcano plots of pairwise comparisons of reperfused or non-ischemic lobes compared to pooled shams. To identify differentially expressed genes, filtered probe sets corresponding to coding genes (11,704) were subjected to one-way ANOVA. The comparisons were pairwise for either the reperfused or non-ischemic lobes at each time versus the results of pooled sham-operated animals. There were little changes in gene expression in either the reperfused or non-ischemic lobes directly after ischemia (0 hr). After 0.5 hr of reperfusion, plots show probe sets with highly significant fold-changes in both the non-ischemic and reperfused lobes, with 33.6% overlap in the genes whose changes were significant. After 2 and 6 hr of reperfusion, the volcano plots showed similar transcriptional patterns. The significant genes at both reperfusion times showed considerable overlap between the reperfused and non-ischemic comparisons (60.1% and 69.1% of probe sets in the 2 hr and 6 hr groups, respectively).

To further determine the differences between the reperfused and non-ischemic lobes at each time point, we compared each of these lobes to the pooled shams (Fig 5). After thirty minutes of ischemia and before reperfusion (0 hr), there were minimal significant changes in gene expression in either lobe compared to sham. Only two genes, Cox8a and Fos, were found to be differentially expressed in the reperfused comparison. In the non-ischemic comparison, 34 annotated genes were found to change significantly. However, there were numerous probe sets that were significantly up and downregulated in either lobe compared to sham at the other reperfusion times. Strikingly, there was a considerable degree of overlap between significant probe sets in the reperfused and non-ischemic lobes when either was compared to the sham group (S2 Table). The proportion of differentially expressed probe sets in the reperfused lobes that were also present in the non-ischemic lobes was 33.6%, 60.1%, and 69.1%, at 0.5, 2 and 6 hr of reperfusion, respectively.

The volcano plot of the 0.5 hr reperfused lobes showed more genes with a high fold change at this earliest reperfusion time relative to the other two reperfusion times. In the 0.5 hr comparison, there were over 20 genes with fold-changes greater than 10.0, while the maximum fold change at 2 and 6 hr of reperfusion were 5.1 and 4.8, respectively. The probe sets that changed significantly relative to the sham group differed at each time point, indicating that changes in gene expression were not sustained across the reperfusion time course. We interpreted this as indicating that multiple stimuli and signaling events mediate the changes in gene expression during the early phase of reperfusion.

### Pathway analysis identifies discrete upstream regulation of gene expression during early reperfusion

To further examine the discrete temporal hepatocellular responses to transient IR, we performed Ingenuity Pathway Analysis (IPA®) of pairwise comparisons of the reperfused versus ischemic lobes and each lobe compared to sham at each time point. Significant probe sets (q-value < 0.05) derived from the pairwise ANOVA were used. For both Canonical Pathway and Upstream Analysis analyses, three different sets of genes were analyzed separately, all significant genes regardless of direction of change, upregulated genes and downregulated genes. S3 Table contains results for the combined gene set for Canonical Pathway analyses and the separate analyses for upregulated and downregulated genes for Upstream Analysis. All three sets of genes yielded similar results in terms of significant pathways and upstream regulators. Comparisons between the reperfused and the non-ischemic lobes at each time point did not yield any significant canonical pathways and only yielded significant results in the Upstream Analysis at 0.5 hr of reperfusion. However, all of the target gene sets were relatively small in number with the largest set, that belonging to TNF, containing 20 genes (S3 Table).

Canonical Pathway analyses comparing the reperfused or non-ischemic lobes to the pooled shams yielded no significant findings at 0.5 hr and only borderline significant changes at the 2 and 6 hr time points. (S3 Table). At 2 hours, there were only a few pathways found in the reperfused and non-ischemic lobes with p-values close to the threshold of significance. These involved various cellular signaling pathways. At six hours of reperfusion, almost all pathways identified were found in both lobes, including EIF2 Signaling and Protein Ubiquitination. In contrast to Canonical Pathways, upregulated genes for every dataset yielded significant results in the Upstream Regulator analysis (S3 Table). However, upstream regulators identified using the non-ischemic data were limited and showed redundancy in the regulators in the time-matched reperfused lobes, particularly at 2 and 6 hr.

The Upstream Analysis of upregulated genes after 0.5 hours of reperfusion produced the largest dataset, where 296 and 33 upstream regulators were enriched in the reperfused lobes and non-ischemic lobe, respectively. The most highly significant upstream regulators in the reperfused lobe (PDGF BB, IL1B, TNF, IGF1) were based on a relatively limited target gene set that included immediate early genes (ATF3, EGR1, FOS, JUN). These immediate early genes were common to multiple results identified in the Upstream Regulator analysis (S3 Table).

To further evaluate effects on the reperfused lobe, we examined the target gene sets of the top 25 upstream regulators (S3 Table). We matched these results with those found in the accompanying non-ischemic lobe and found 6 were common to both lobes. Notably, each of these common upstream regulators had a higher significance in the reperfused lobes based on a greater number of target genes. We also performed Signosis transcription factor activity arrays to assess differences in the activity of 95 transcription factors in the reperfused lobe compared to sham controls at 0.5 hr of reperfusion. There were no significant changes in activity between the reperfused and sham controls in any of the transcription factors on the array. S1 Fig shows the activity of 25 transcription factors with known roles in the response to ischemia-reperfusion and oxidative stress, including AP-1, HIF, STATs and NFkB. We interpreted these data as indicating that the immediate early gene response may not involve activation of any of these well characterized, canonical transcription factors.

At 2 hr of reperfusion, the upregulated genes led to the identification of 25 upstream regulators in the reperfused lobe and 6 regulators in the non-ischemic lobe. Regulators unique to the reperfused lobes included cytokines, growth factors, and signaling proteins (e.g.,TP53, PDGF BB, TGFB1). These factors were identified based on overlapping target gene sets involved in extracellular matrix maintenance and cytoskeletal remodeling. We interpreted these data as indicating a wound healing-type response at 2 hr of reperfusion that was exclusive to the reperfused lobe.

At 6 hr of reperfusion, there were 44 and 32 upstream regulators identified for the reperfused and non-ischemic lobes, respectively. Twenty-five of these regulators were common to both lobes. These included HNF4A, RICTOR and myc. The target genes accounting for these factors appeared to represent induction of normal metabolic functions relative to the sham group. We hypothesized that these similar transcriptional changes in both lobes at six hours as a global shift toward normal liver metabolic functions. Transcriptional events that occurred at 6 hr of reperfusion differed greatly from those found at 0.5 and 2 hr in two ways. First, unlike the other times, the 6 hr reperfused and non-ischemic lobes produced almost completely overlapping results. Second, the transcriptional response seemed to be driven by changes in genes related to normal metabolism. This response is unlike that found at the earlier time points, where changes in the reperfused lobe appeared to be regulated by factors and pathways usually associated with a damage response.

### Thirty minutes of reperfusion induces immediate early genes and a stress-induced transcriptional response

As noted above, the most marked changes in the reperfused lobes occurred at 0.5 hours of reperfusion. We identified the 25 genes with the largest fold change in expression. The corresponding fold changes and q-values of these 25 genes were matched to those found in the non-ischemic comparison (Table 1). The genes were tabulated into three groups for comparison: genes exclusive to the ischemic lobe, those found in both lobes but with fold changes in the reperfused lobe that were at least twice that in the non-ischemic lobe (shaded in grey), and those with increased expression in both lobes. Notably, none of these 25 enriched genes were identified by ANOVA at either 2 or 6 hours of reperfusion (S2 Table). Examination of the genes exclusive to the reperfused lobe showed that they were consistent with an oxidative stress response (Cyr61, Fosb, Egr1, Gadd45a), chemokine and cytokine signaling (Ccl3, Junb, Cxcl10), and an inflammatory/immune response (Fgf21, Alox15, Ier2). Several immediate-early response genes (Fos, Jun, Atf3, Egr2) were found in both lobes, but had significantly higher fold-changes in the reperfused lobes. RT-qPCR was performed on several of these genes to confirm the changes observed in the reperfused compared to non-ischemic lobe (S2 Fig). Examination of our IPA results showed that these 25 genes contributed substantially to the identification of multiple upstream regulators in the reperfused lobe. This subset of genes also contributed to the higher level of significance in the reperfused lobe compared to the non-ischemic lobe seen for IPA results common to both groups. We interpret these results as indicating that those differentially expressed genes exclusive to the ischemic lobe could be a result of an IR-mediated response autonomous to the hepatic parenchyma, while genes whose expression was affected in both the non-ischemic and reperfused lobes may be a result of changes in circulating factors or hemodynamics changes associated with interruption of flow to the ischemic-reperfused lobes [30–32].

**Table 1:** Results for the 25 genes with the greatest fold change in the reperfused and non-ischemic lobes relative to the sham surgery group after thirty minutes of reperfusion.

		Reperfused			Non-Ischemic		
Gene Symbol	Gene Name	Fold Change	log2 Fold Change	q-value	Fold Change	log2 Fold Change	q-value
Cyr61	Cysteine Rich Angiogenic Inducer 61	94.56	6.56	1.26E-04	---	---	---
Egr1	Early Growth Response 1	89.14	6.48	0.007	---	---	---
Ier2	Immediate Early Response 2	49.60	5.63	2.30E-06	---	---	---
Fosb	FosB Proto-Oncogene, AP-1 Transcription Factor Subunit	45.24	5.50	1.82E-16	---	---	---
Ccl3	C-C Motif Chemokine Ligand 3	17.35	4.12	0.024	---	---	---
Fgf21	Fibroblast Growth Factor 21	16.90	4.08	0.008	---	---	---
Junb	JunB Proto-Oncogene, AP-1 Transcription Factor Subunit	12.25	3.62	2.41E-06	---	---	---
Cxcl10	C-X-C Motif Chemokine Ligand 10	9.99	3.32	0.003	---	---	---
Gadd45a	Growth Arrest And DNA Damage Inducible Alpha	9.85	3.30	2.25E-04	---	---	---
Alox15	Arachidonate 15-Lipoxygenase	9.74	3.28	0.024	---	---	---
Fos	Fos Proto-Oncogene, AP-1 Transcription Factor Subunit	1823.49	10.83	2.77E-14	17.01	4.09	3.13E-07
Jun	Jun Proto-Oncogene, AP-1 Transcription Factor Subunit	102.91	6.69	5.74E-04	23.16	4.53	0.016
Gdf15	Growth Differentiation Factor 15	68.85	6.11	5.66E-13	6.26	2.65	1.14E-05
Atf3	Activating Transcription Factor 3	61.97	5.95	1.85E-10	3.56	1.83	0.001
Rgs1	Regulator Of G Protein Signaling 1	26.48	4.73	9.74E-08	13.52	3.76	5.05E-05
Egr2	Early Growth Response 2	21.42	4.42	4.31E-07	1.62	0.70	0.029
Rhob	Ras Homolog Family Member B	21.15	4.40	9.07E-04	7.27	2.86	0.030
Zfp36	ZFP36 Ring Finger Protein	13.58	3.76	1.87E-07	2.60	1.38	0.029
Nfkbiz	NFKB Inhibitor Zeta	13.09	3.71	1.85E-08	3.83	1.94	0.034
Btg2	BTG Anti-Proliferation Factor 2	43.93	5.46	0.003	33.40	5.06	0.008
Apold1	Apolipoprotein L Domain Containing 1	33.28	5.06	2.77E-09	18.50	4.21	1.56E-06
Nr4a1	Nuclear Receptor Subfamily 4 Group A Member 1	24.30	4.60	7.61E-04	47.34	5.56	6.19E-05
Tnfaip3	TNF Alpha Induced Protein 3	17.85	4.16	0.001	10.69	3.42	0.009
Ppp1r15a	Protein Phosphatase 1 Regulatory Subunit 15A	16.41	4.04	3.90E-04	10.62	3.41	0.004
Serpine1	Serpin Family E Member 1	11.14	3.48	0.009	17.54	4.13	0.002

Grey shading indicates those genes that were induced in both the reperfused and non-ischemic lobes.

### Cluster analysis of temporal changes in genes during early reperfusion

**Fig 6:**
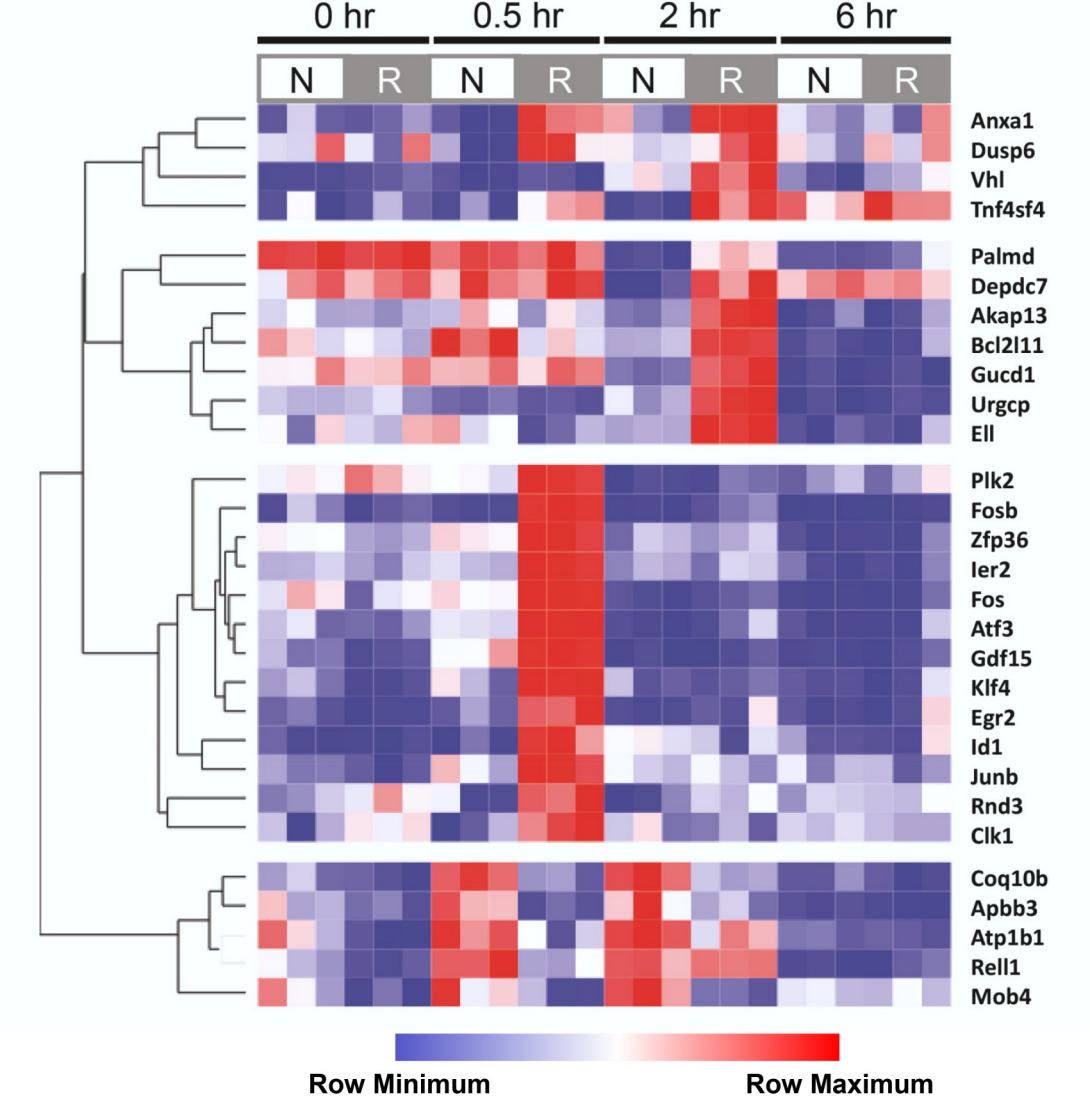
Cluster analysis of the temporal changes in gene expression during early reperfusion. One-way ANOVA was performed across all probe sets (11,704) for the reperfused and non-ischemic samples (n = 3) at all reperfusion times. The resulting 29 significant probe sets (q< 0.05) were used to construct a heat map with hierarchical clustering by row (Pearson’s correlation). Four well-defined gene clusters are shown. The intensity of color corresponds to highest (red) and lowest (blue) expression for that gene relative to the mean expression for that gene (white).

To further investigate the temporal pattern of gene expression in the reperfused and non-ischemic lobes, we performed one-way ANOVA on all 11,704 probe sets. Using a significance threshold of q<0.05, 29 probe sets, corresponding to annotated genes, were identified for inclusion in a heat map. We performed hierarchical clustering by row to discern temporal patterns of expression (S4 Table). Four well-defined clusters of annotated genes emerged (Fig 6). The first two clusters showed a marked increased expression in only the ischemic group after 2 hr of reperfusion. The first cluster consisted of 4 genes (Tm4sf4, Anxa, Dusp6, Vhl) linked to cell adhesion and regulation of cell shape, while the second cluster contained 7 genes involved in cell survival and general signal transduction (including Palmd, Akap13, Gucd1). Cluster 3 was comprised of genes with increased expression in the ischemic group after 0.5 hr of reperfusion. This cluster consisted of 13 genes, including immediate-early response genes and those associated with a HIF-1α-independent gene response [33]. The genes in cluster 3 had, in general, much higher expression than any other genes displayed in the heatmap (S4 Table). Cluster 4 showed a relative increase in expression in the non-ischemic lobe at 0.5 and 2 hr of reperfusion. The five genes in cluster 4 were associated with liver metabolic function.

### Phosphorylation events in the reperfused lobes correspond to oxidative stress-mediated wound healing

To investigate early signal transduction events that might be involved in the transcriptomic response to reperfusion, we performed high-throughput protein phosphorylation screening. We used Kinexus antibody microarrays in a comparison of the reperfused and non-ischemic lobes. We focused on phosphorylation events after two hours of reperfusion, reasoning that short term changes would likely persist at this time point. Among the 880 phosphosites on the arrays, a total of 15 had an FDR <0.05, with 13 upregulated and 2 downregulated in the reperfused lobes compared to the non-ischemic lobes (S5 Table). The majority of the identified phosphosites represented activating phosphorylation events, as indicated by examination of the PhosphoSitePlus® and UniProt knowledge bases [34, 35]. These markers of signaling events, including p70S6K, Paxillin1, CDK1, and Cofilin1, have been implicated in pathways regulating cell adhesion, cytoskeletal reorganization and cell survival. Some phosphosites showed large fold-changes but did not achieve statistical significance based on biological variability within groups. Activating phosphorylation sites on Jun (Y170) and Fos (T232) were increased in the reperfused lobe, but did not reach statistical significance. Notably absent were changes in the phosphorylation state of proteins in the Raf-MEK-Erk signaling cascade.

We went on to examine changes in the activity of JNK, ERK1/2 and p38 signaling in the reperfused and non-ischemic lobes following 30 minutes of reperfusion (S3 Fig). We did not observe any significant differences in the phosphorylation of these signaling components at any of the reperfusion time points analyzed. We also examined AMPK and mTORC1 signaling in the reperfused and non-ischemic lobes as these two pathways are responsive to fluctuations in energy and nutrients levels [11]. In addition, these pathways play a role in regulating autophagy which is known to be important for cell survival following ischemia-reperfusion injury [36]. We did not observe changes in AMPK or mTORC1 signaling in the reperfused compared to non-ischemic lobe (S4 Fig).

## Discussion

Current targeted therapies under investigation to attenuate hepatic ischemia-reperfusion (IR) injury aim to blunt the progression of early reperfusion, and the resulting damage, through the inhibition of pathways induced by reactive oxygen species (ROS) and inflammation [9]. However, there has been limited success in the clinical translation of targeted interventions found to ameliorate hepatic damage in animal models of IR injury. Recent literature challenges this canonical oxidative stress-driven IR injury paradigm [13, 37, 38]. Rather, these studies, which employed an omics approach, suggest that newly-identified signaling pathways promote injury in IR models.[14] These pathways, including dysregulated lipid metabolism, are shown to be critical and sufficient to induce cell dysfunction and death in IR injury models. These findings suggest further investigation is necessary to understand mechanistic responses to ischemia which, in addition to metabolic changes and hypoxia, includes stimuli associated with shear stress, altered nutrient availability and humoral factors.

In contrast to targeted therapies, ischemic preconditioning (IPC) is a therapeutic approach that reduces IR-mediated damage by stimulating an adaptive response prior to injury [39]. While IPC is effective in reducing hepatic damage in preclinical IR injury models, clinical translation has yielded conflicting results [15–18]. The protective mechanisms of IPC have been characterized extensively to include increased antioxidant activity, decreased ROS production, and decreased neutrophil infiltration. Recently, COX-2, a prostaglandin-endoperoxide synthase that is upregulated during inflammation, has been identified as a protective mediator induced by IPC in hepatocytes in an IR injury model [14]. Profiling signaling pathways and their upstream regulation stimulated by transient ischemia can produce more refined targets to mitigate IR-mediate damage to test in patient trials.

In this study, we examined a model of hepatic IR and found that many of the changes in gene expression that could be attributed to IR were also present in the lobes unaffected by ischemia.

Our experimental design used a well-established 70% warm transient ischemia-reperfusion rat model to compare changes in gene expression in the reperfused lobes to the other 30% of liver (non-ischemic lobes) that did not experience blood interruption [40, 41]. To our knowledge, this is the first study to profile the transcriptome of the non-ischemic lobe in any warm 70% IR model. These changes were compared to sham-operated animals at four time points: directly after 30 minutes of ischemia and at 3 reperfusion durations selected to profile early events. We also selected a duration of ischemia intended to induce signaling events and transcriptomic changes without overt injury. Our experimental design offered three potential benefits. First, with a brief ischemic period, we could profile the molecular mechanisms that govern cell responses to transient ischemia that are independent of hepatic necrosis and immune cell recruitment. This allowed us to examine early responses by the resident cell population without the confounding factors of an inflammatory response or cell death. Second, we could account for changes in hemodynamics and humoral factors to identify responses exclusive to IR by profiling both the reperfused and non-ischemic lobes and comparing them to sham-operated animals. Lastly, by examining several times of reperfusion, we could compare transcriptional changes at the onset, middle, and end of the early reperfusion phase.

At all three reperfusion times, there were hundreds of genes that changed significantly in the reperfused lobe when compared to the sham group. However, while the transcriptomes of the reperfused lobes differed from one another at each of the three reperfusion times, they were strikingly similar to their time-matched non-ischemic counterparts. One explanation for this observation is that many of the transcriptional events that occur during early reperfusion do not represent an autonomous response in the reperfused lobe. Rather, they may be dependent on circulating factors that are released from the ischemic-reperfused lobe and affect the whole of the liver. We also considered the possibility that these tissue non-autonomous events represent changes in hemodynamics. The changes in blood flow to the non-ischemic lobe when flow to the ischemic lobe is interrupted may involve a transient increase that produces congestion and, as a result, impaired flow. Regardless, the global liver response is specific to IR, as the changes were not seen in the sham-operated animals.

Pathway analyses did not reveal convincing evidence of the activation of specific canonical transcription factors at any time point. Subsequent phosphorylation screening also did not implicate a specific pathway that could explain this transcriptional profile. We intend to undertake a more complete analysis of signaling events using mass spectrometry based phosphoproteomics. That approach was beyond the scope of the present study.

The briefest period of reperfusion that we examined (0.5 hr) had the largest set of differentially expressed genes. Thirty minutes of reperfusion was enough to induce marked increases in genes that together form an immediate-early gene response unique to the reperfused lobe. At this time point, the reperfused lobes were the most unlike their non-ischemic counterparts, with over 70% of changes found exclusively in the reperfused lobe. This earliest response found in the reperfused lobe consisted of a set of immediate early genes (Atf3, Egr1, Fos, Fosb, Jun, Junb) and those that encode chemokines and other secreted proteins (Ccl3, Cxcl10, Ier2, Fgf21 All of these genes had dramatic fold-changes, ranging from 10-fold to over 1000-fold). Many of these genes have been implicated in the response to ischemia reperfusion in brain and heart [42, 43]. In these models, the activation of immediate early genes is correlated with cell proliferation and differentiation, while chemokines are associated with inflammation. Fgf21 is a secreted protein whose main site of production is the liver. The serum level of Fgf21 has been shown to be a prognostic biomarker of the severity of ischemia reperfusion injury in patients with liver transplantation [44]. However, the damage only occurs after the marked increase in circulating Fgf21 are attenuated, suggesting that FGF21 may have a role in protection. Fgf21 treatment of cultured cardiomyoctes induced the expression of genes involved in antioxidant pathways [45]. Similarly, Fgf21 has been shown to promote functional recovery in a model of neonatal hypoxia-ischemia [46]. Fgf21 has also been shown to prevent liver injury in mouse models of ethanol binge drinking [47]. The studies raise the possibility that Fgf21 production in the liver may be upregulated in response to stress in order to provide a protective effect. The precise role of immediate early genes and secreted proteins, such as, chemokines and Fgf21 in hepatic ischemia reperfusion remain unknown and represent intriguing targets for further study.

Unlike the 0.5 hr groups, the reperfused and non-ischemic lobes at 2 and 6 hours of reperfusion showed a marked overlap in expression changes. Over 60% of the affected genes in the reperfused lobe were also affected in the non-ischemic at the 2 and 6 hr time points. However, there was one notable change at the 2 hr time point that was specific to the reperfused lobe. After 2 hr of reperfusion, genes involved in a wound healing-type damage response were upregulated in the reperfused lobe only. This damage response did not persist at 6 hr.

Our analyses were performed on liver tissue. However, as hepatocytes constitute 70% of liver mass, our data most likely reflect changes in the hepatic transcriptome. Hepatocyte-derived signaling could act as a rheostat for IR responses, including inflammatory signaling, neutrophil infiltration, cell survival and cell death. We originally hypothesized that in our model, the brief period of ischemia would induce multiple cell signaling pathways that would promote cell survival [48, 49]. This hypothesis was partly based on previous studies where targeted interventions, such as inhibition of mTORC1 [11], were used to alleviate hepatic damage. However, our results indicate that canonical signaling pathways, such as, mTORC1 and MAPK signaling and anti-oxidant responses cannot account for the complex and broad changes in the transcriptome we observed, especially at the earlier time points of reperfusion.

Finally, the absence of data pointing to roles for specific transcription factors or signaling networks that would account for these changes in gene expression may indicate non-canonical signaling is involved in these early events. One possibility is an alteration in energy metabolism that induces changes in local metabolite concentrations, histone modifications, and chromatin structure. Changes in the epigenome in response to liver ischemia reperfusion has not been previously investigated and has great potential for modulating gene expression and pathways involved in tissue response to injury. This is an area that will come under investigation as we pursue further studies.

## Supporting information

S1 Fig Transcription factor (TF) activity in the 0.5 hr reperfused and sham groups. Signosis transcription factor arrays were performed on nuclear extracts isolated from triplicate reperfused lobes 30 minutes following reperfusion and sham controls. The graph depicts TF activity (relative fluoresecence units; RFUs) for 25 TFs with known roles in the response to oxidative stress. Data are shown as mean + 1 SD.
(TIF)

S2 Fig Relative expression of *Cyr61*, *Fgf21* and *Fos* in the reperfused compared to non-ischemic lobes at 0.5 hr of reperfusion. Relative mRNA expression levels for *Cyr61*, *Fgf21* and *Fos* were determined by RT-qPCR. Each gene of interest was quantified using the comparative CT method with *18s* as a reference. Fold-change (the ratio of the reperfused to non-ischemic lobes) for each animal are shown as individual points with the line indicating the mean of the triplicate measurements.
(TIF)

S3 Fig Representative images of western immunoblots for three MAPKs. Phosphospecific western immunoblotting was performed to assess the activity of three MAPKs, JNK, ERK and p38. Antibodies directed toward the non-phosphorylated, total protein were used to assess differences in stoichiometry of phosphorylation (the ratio of phospho- to total). The analysis was performed on quadruplicate biological replicates of the non-ischemic (“N”) and reperfused (“R”) lobes. An EGF/insulin treated sample (“C”) were added to each blot as a positive control for MAPK activation. Representative immunoblots from samples obtained at 0.5 hr of reperfusion are shown. **(A)** Phospho-ERK 1/2 (T202/Y204) and total ERK 1/2. **(B)** phospho-JNK (T183/Y185) and total JNK. **(C)** Phospho p-p38 (T180/Y182) and total p38. No statistically significant changes in the phospho/total ratios between the reperfused and non-ischemic lobes were observed for ERK, JNK or p38. The total blots for each protein were stripped and reprobed for GAPDH.
(TIF)

S4 Fig Representative images of western immunoblots representing AMPK/mTORC1 activities. Western immunoblot analysis was performed on quadruplicate biological replicates of the non-ischemic (“N”) and reperfused (“R”) lobes at 0.5h of reperfusion and a positive control using an EGF/insulin treated sample (“C”) were added to each blot. **(A)** Phospho-AMPKα (T172) and total AMPKα at 30 minutes of reperfusion. **(B)** Phospho-S6 (S235/S236) and total S6 immunoblots at 30 minutes of reperfusion. No statistically significant changes in the phospho/total ratios between the reperfused and non-ischemic lobes were observed for AMPK or S6. The total blots for each protein were stripped and reprobed for GAPDH.
(TIF)

S5 Fig Unadjusted images of western immunoblots shown in supplemental S3 and S4 Figs. Western immunoblot analysis was performed on quadruplicate biological replicates of the non-ischemic (“N”) and reperfused (“R”) lobes at 0.5h of reperfusion and a positive control using an EGF/insulin treated sample (“C”) were added to each blot. Pre-stained molecular weight markers are labeled as M.
(PDF)

S1 Table Histologic scoring of H&E sections. Multiple 20x sections of the non-ischemic and reperfused lobes from duplicate animals were scored using a modified Suzuki scale by a blinded pathologist.
(XLSX)

S2 Table Significant probe sets in pairwise comparisons of reperfused versus non-ischemic lobes and reperfused or non-ischemic lobes versus pooled shams. Probe sets (11,704) were used for pairwise ANOVA. Statistically significant probe sets (q<0.05) are listed with probe id, gene symbol, log_2_ fold-change, and q-value. Each worksheet represents a comparison of two specific groups at one time point.
(XLSX)

S3 Table Significant probesets from the pairwise comparison of the reperfused versus non-ischemic lobes across all reperfusion times. Probe sets (11,704) were used for pairwise ANOVA. Statistically significant probe sets (q<0.05) are listed with probe id, gene symbol, and expression value for each biological replicate.
(XLSX)

S4 Table Significant IPA results from the pairwise comparisons of reperfused versus non-ischemic and reperfused or non-ischemic lobes versus pooled shams. Differentially expressed probe sets from ANOVA of pairwise comparisons were used for IPA (Core Analysis). Combined gene sets were analyzed for Canonical Pathway while upregulated and downregulated gene sets were analyzed separately for Upstream Analysis. All gene sets yielded similar results. The combined gene sets for each analysis are shown. Top results from the Upstream Analysis found in the 0.5 hr reperfused comparison were compared to that found in the 0.5 hr non-ischemic counterpart.
(XLSX)

S5 Table Kinexus results. We performed pairwise comparisons of reperfused or non-ischemic lobes compared to pooled shams. Protein homogenates from reperfused and non-ischemic lobes at 2 hr of reperfusion as well as pooled shams were used for Kinexus KAM 900P antibody arrays. Raw data values are displayed in colored cells that is based on a color gradient; 0 is deep blue, the median value is white, and 100,000 is red. Statistically significant results from the one-way ANOVA results are tabulated with fold changes, p-values, and q-values.
(XLSX)

## Abbreviations

ALT: alanine aminotransferase
ANOVA: analysis of variance
AST: aspartate aminotransferase
FDR: false discovery rate
H&E: hematoxylin and eosin
hr: Hour
IPA: Ingenuity Pathway Analysis
IPC: ischemic preconditioning
IR: ischemia-reperfusion
PC: principal component
PCA: principal component analysis
ROS: reactive oxygen species
