# Supplementary figures and images for: Transcriptional changes during hepatic ischemia-reperfusion in the rat
Source: PLoS One. 2019 Dec 31;14(12):e0227038. doi: 10.1371/journal.pone.0227038 (PMC6938360; doi:10.1371/journal.pone.0227038)

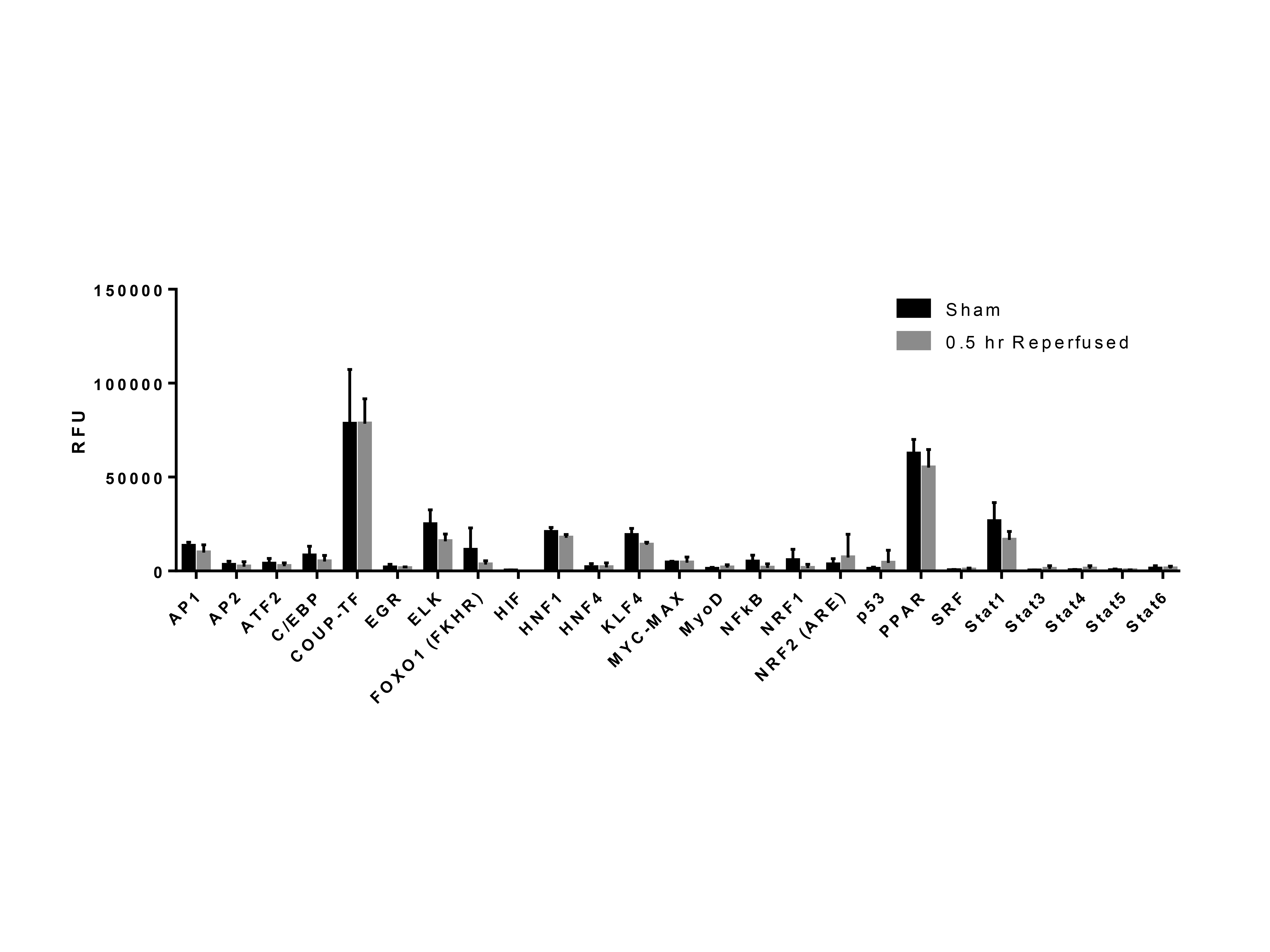

Supplement: S1 Fig — Signosis transcription factor arrays were performed on nuclear extracts isolated from triplicate reperfused lobes 30 minutes following reperfusion and sham controls. The graph depicts TF activity (relative fluoresecence units; RFUs) for 25 TFs with known roles in the response to oxidative stress. Data are shown as mean + 1 SD. (TIF) [file pone.0227038.s001.tif]

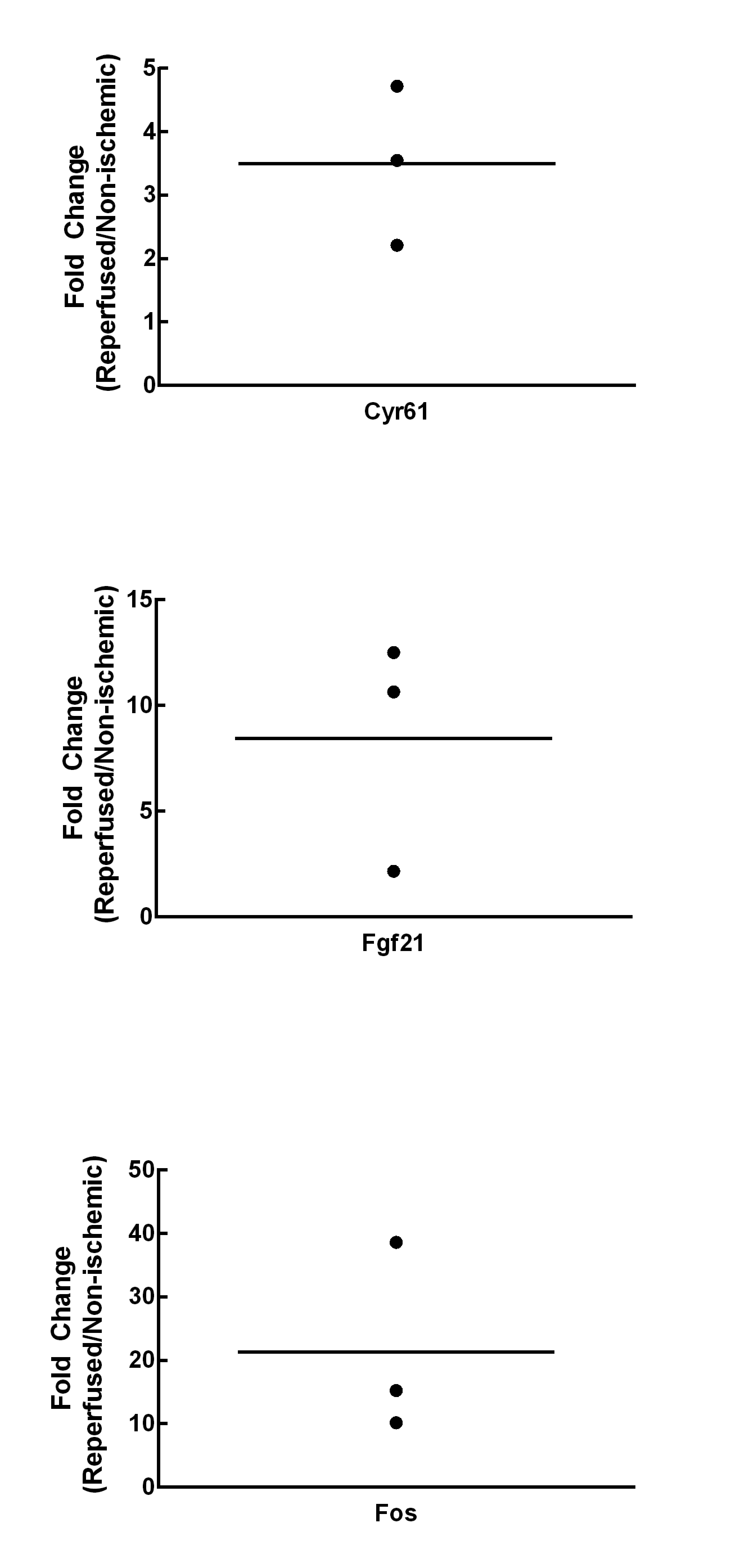

Supplement: S2 Fig — Relative mRNA expression levels for Cyr61, Fgf21 and Fos were determined by RT-qPCR. Each gene of interest was quantified using the comparative CT method with 18s as a reference. Fold-change (the ratio of the reperfused to non-ischemic lobes) for each animal are shown as individual points with the line indicating the mean of the triplicate measurements. (TIF) [file pone.0227038.s002.tif]

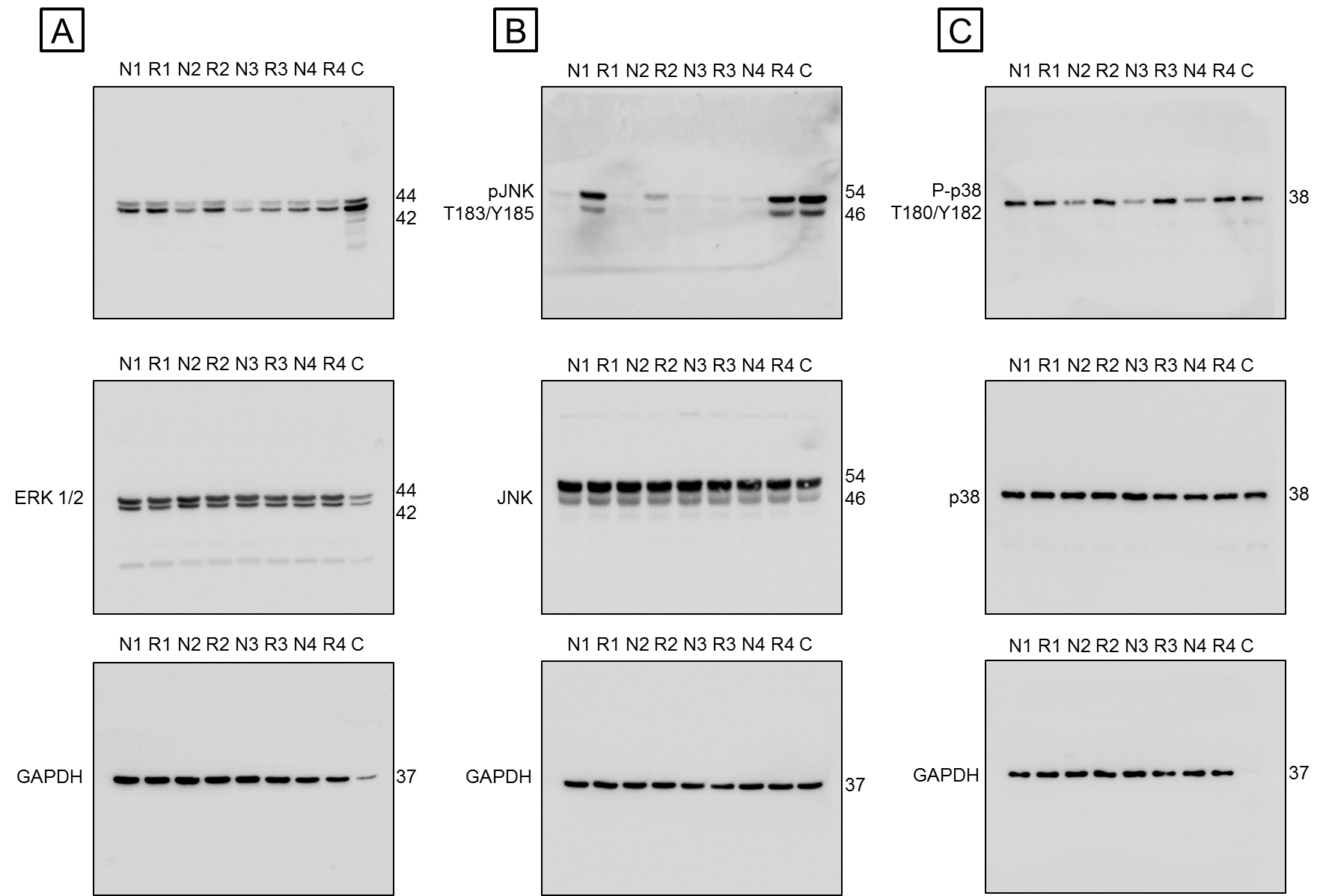

Supplement: S3 Fig — Phosphospecific western immunoblotting was performed to assess the activity of three MAPKs, JNK, ERK and p38. Antibodies directed toward the non-phosphorylated, total protein were used to assess differences in stoichiometry of phosphorylation (the ratio of phospho- to total). The analysis was performed on quadruplicate biological replicates of the non-ischemic (“N”) and reperfused (“R”) lobes. An EGF/insulin treated sample (“C”) were added to each blot as a positive control for MAPK activation. Representative immunoblots from samples obtained at 0.5 hr of reperfusion are shown. (A) Phospho-ERK 1/2 (T202/Y204) and total ERK 1/2. (B) phospho-JNK (T183/Y185) and total JNK. (C) Phospho p-p38 (T180/Y182) and total p38. No statistically significant changes in the phospho/total ratios between the reperfused and non-ischemic lobes were observed for ERK, JNK or p38. The total blots for each protein were stripped and reprobed for GAPDH. (TIF) [file pone.0227038.s003.tif]

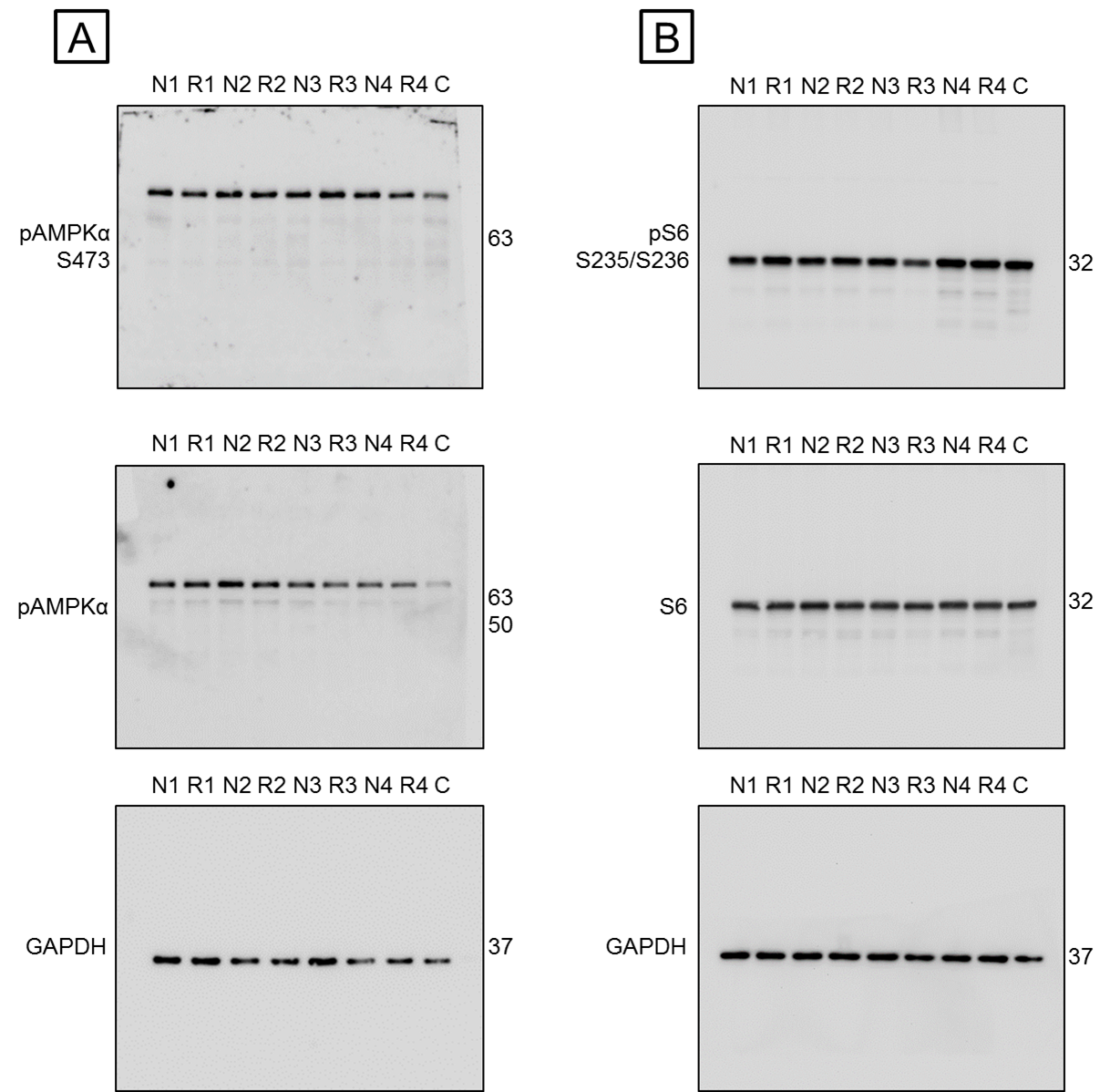

Supplement: S4 Fig — Western immunoblot analysis was performed on quadruplicate biological replicates of the non-ischemic (“N”) and reperfused (“R”) lobes at 0.5h of reperfusion and a positive control using an EGF/insulin treated sample (“C”) were added to each blot. (A) Phospho-AMPKα (T172) and total AMPKα at 30 minutes of reperfusion. (B) Phospho-S6 (S235/S236) and total S6 immunoblots at 30 minutes of reperfusion. No statistically significant changes in the phospho/total ratios between the reperfused and non-ischemic lobes were observed for AMPK or S6. The total blots for each protein were stripped and reprobed for GAPDH. (TIF) [file pone.0227038.s004.tif]
