# Supplementary material for: Transcriptional changes during hepatic ischemia-reperfusion in the rat
Source: PLoS One. 2019 Dec 31;14(12):e0227038. doi: 10.1371/journal.pone.0227038 (PMC6938360; doi:10.1371/journal.pone.0227038)
Supplement: S5 Fig — Western immunoblot analysis was performed on quadruplicate biological replicates of the non-ischemic (“N”) and reperfused (“R”) lobes at 0.5h of reperfusion and a positive control using an EGF/insulin treated sample (“C”) were added to each blot. Pre-stained molecular weight markers are labeled as M. (PDF) [file pone.0227038.s005.pdf]

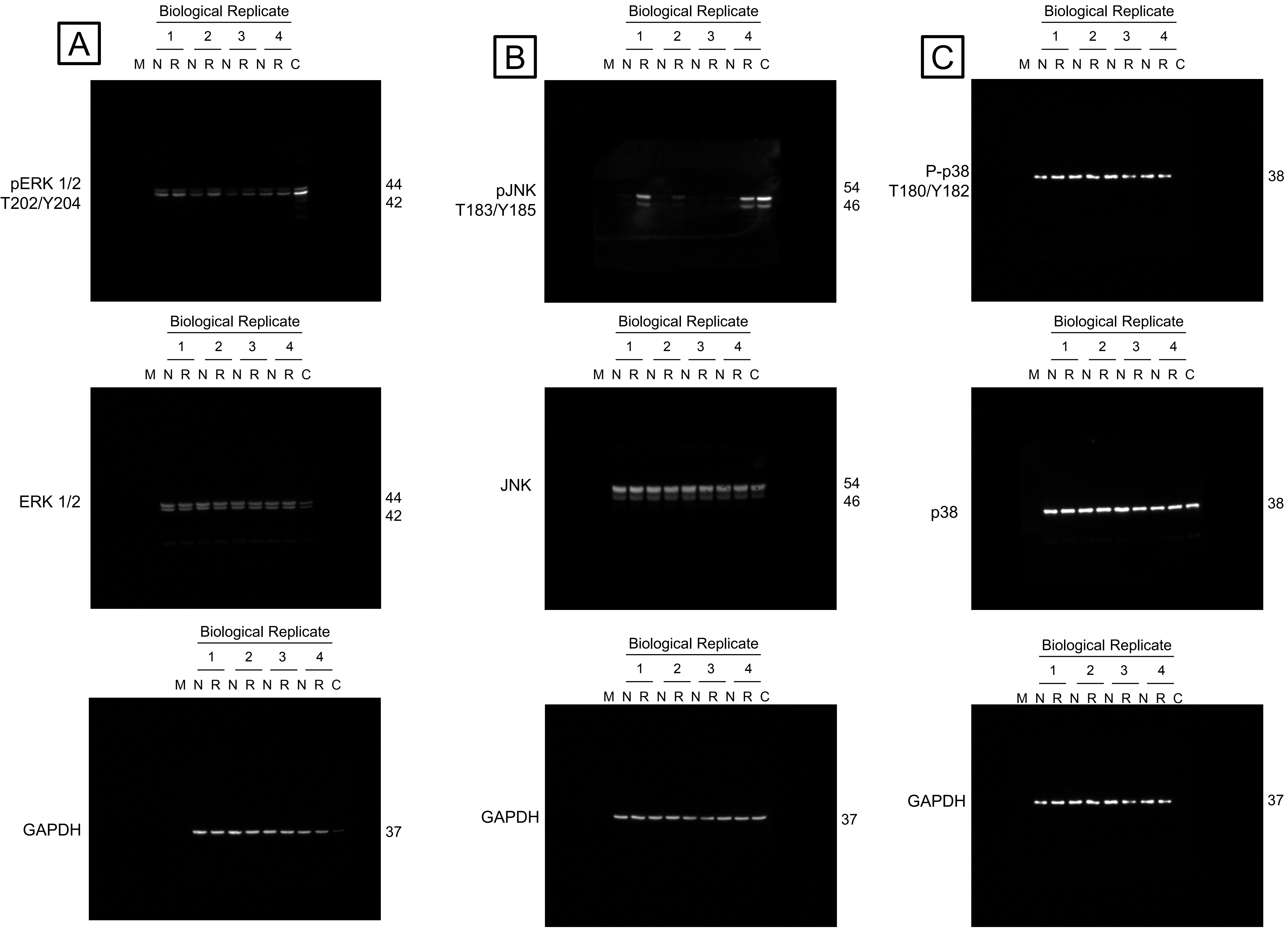
A
B
C
Biological Replicate
1 2 3 4
M N R N R N R N R C
pERK 1/2 T202/Y204
44
42
ERK 1/2
44
42
GAPDH
37
pJNK T183/Y185
54
46
JNK
54
46
GAPDH
37
P-p38 T180/Y182
38
p38
38
GAPDH
37

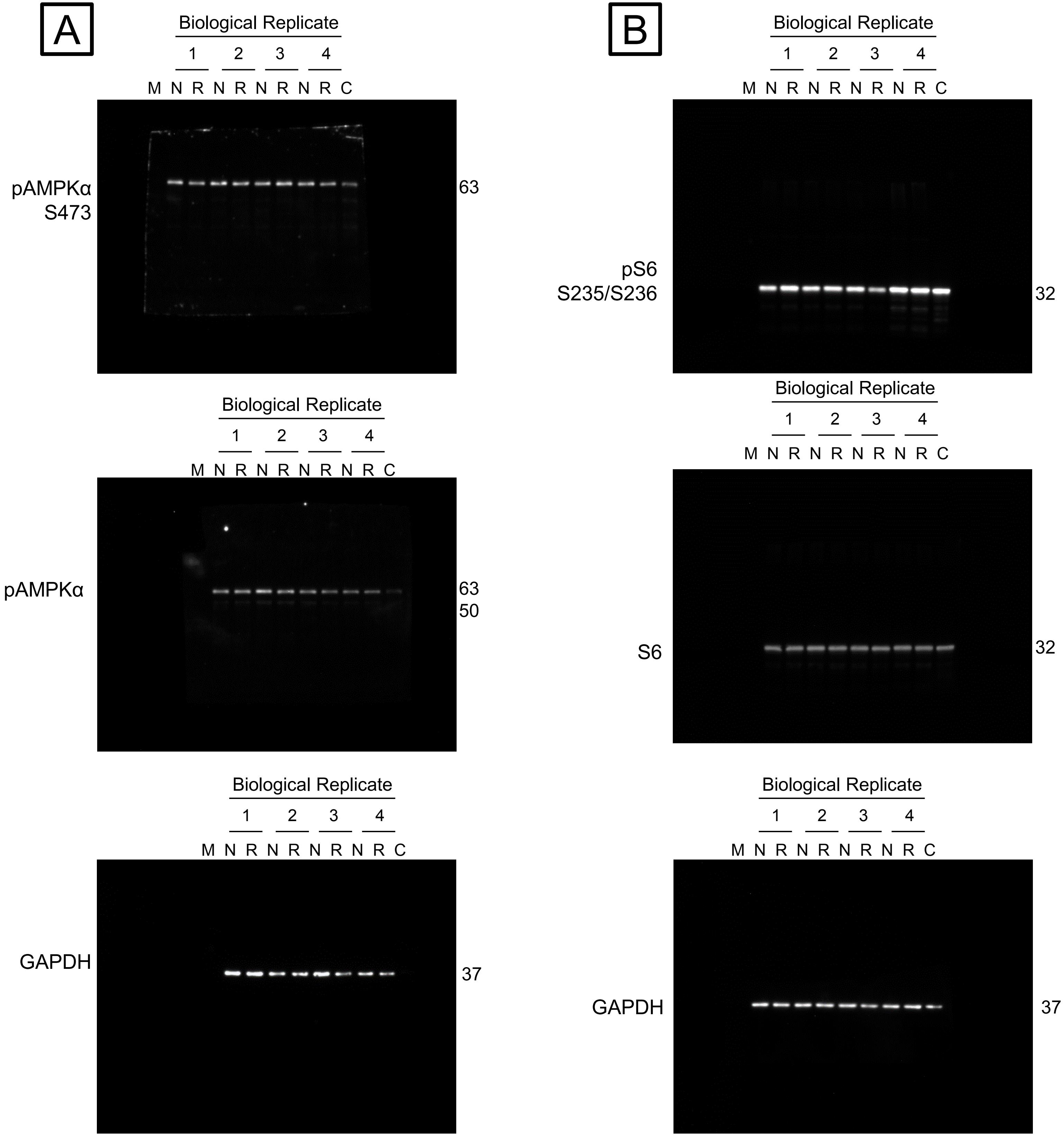
A
Biological Replicate
1 2 3 4
M N R N R N R N R C
pAMPKα S473
63
Biological Replicate
1 2 3 4
M N R N R N R N R C
pAMPKα
63
50
Biological Replicate
1 2 3 4
M N R N R N R N R C
GAPDH
37
B
Biological Replicate
1 2 3 4
M N R N R N R N R C
pS6 S235/S236
32
Biological Replicate
1 2 3 4
M N R N R N R N R C
S6
32
Biological Replicate
1 2 3 4
M N R N R N R N R C
GAPDH
37
